# Genotype-specific neoplastic risk profiles in patients with VHL disease

**DOI:** 10.1530/ERC-24-0260

**Published:** 2025-04-28

**Authors:** Athina Ganner, Alfonso Massimiliano Ferrara, Peggy Sekula, Francesca Schiavi, Julia H Joo, Gabriela Sanso, Madson Q Almeida, Anna Laura Knoblauch, Christine Julia Gizaw, Karol Krzystolik, Sophie Charlotte Astheimer, Maria Isabel Achatz, Ana Vieites, Diane Donegan, Thomas Hundsberger, Jan Lubinski, Ilgin Yildirim Simsir, Tushar Bandgar, Kornelia Hasse-Lazar, Agnieszka Pawlaczek, Wouter Zandee, Kai Yu, Claudio E Kater, Liliya Rostomyan, Xiao-Ping Qi, Timo Deutschbein, Hanna Remde, Tabatha Nakakogue Dallagnol, Marina Yukina, Rene Baudrand, Corina E Andreescu, Tada Kunavisarut, Nur Diana Ishak, Xavier Le Guillou Horn, Gemma Shutler, Milan Jovanovic, Mariola Pęczkowska, Jan Calissendorff, Francesco Circosta, Maria João Bugalho, Eleonora P M Corssmit, Oliver Gimm, Marcus Quinkler, Andrea Goldmann, Sara Watutantrige Fernando, Stefania Zovato, Lucas S Santana, Felipe Freitas-Castro, Christian Rothermundt, Josa Zimmermann, Asude Durmaz, Ayca Aykut, Laurent Vroonen, Tobias Krauss, Christian Taschner, Juri Ruf, Jan-Helge Klingler, Sven Gläsker, Stefan Lang, Felicitas Bucher, Hansjürgen Agostini, Cordula Jilg, Wolfgang Schultze-Seemann, Birke Bausch, Antonia Bergfeld, Kilian Rhein, Thomas Uslar, Antonio Concistrè, C Christofer Juhlin, José Cláudio Casali-da-Rocha, Luigi Petramala, Uliana Tsoy, Elena Grineva, Xu-Dong Fang, Fruzsina Kotsis, Tobias Schaefer, Thera P Links, Özer Makay, Gustavo F C Fagundes, Joanne Ngeow, Nalini Shah, Giuseppe Opocher, Marta Barontini, Catharina Larsson, Andrzej Januszewicz, José Viana Lima, Nelson Wohllk, Claudio Letizia, Gianluca Donatini, Eamonn R Maher, Dmitry Beltsevich, Irina Bancos, Cezary Cybulski, Martin K Walz, Anna Köttgen, Charis Eng, Hartmut P H Neumann, Elke Neumann-Haefelin

**Affiliations:** ^1^ Renal Division, Department of Medicine, Medical Center – University of Freiburg, Faculty of Medicine, University of Freiburg, Freiburg, Germany; ^2^ Familial Cancer Clinics, Veneto Institute of Oncology IOV – IRCCS, Padua, Italy; ^3^ Institute of Genetic Epidemiology, Faculty of Medicine and Medical Center – University of Freiburg, Freiburg, Germany; ^4^ Genomic Medicine Institute, Lerner Research Institute; Department of Medical Genetics and Genomics, Medical Specialties Institute; Taussig Cancer Institute, Cleveland Clinic; and Cleveland Clinic Lerner College of Medicine, Cleveland, Ohio, USA; ^5^ Centro de Investigaciones Endocrinológicas “Dr César Bergadá” (CEDIE) CONICET – FEI – División de Endocrinología, Hospital de Niños “Ricardo Gutiérrez”, Buenos Aires, Argentina; ^6^ Laboratório de Endocrinologia Molecular e Celular LIM/25, Divisão de Endocrinologia e Metabologia, Hospital das Clínicas, Faculdade de Medicina da Universidade de São Paulo, São Paulo, Brazil; ^7^ Department of Neurosurgery, Faculty of Medicine, Albert-Ludwigs-University, Freiburg, Germany; ^8^ Department of Ophthalmology, Ministry of Internal Affairs and Administration Hospital, and The International Hereditary Cancer Centre, Szczecin, Poland; ^9^ Department of Urology, Faculty of Medicine, Albert-Ludwigs-University, Freiburg, Germany; ^10^ Centro de Oncologia, Hospital Sírio-Libanês, São Paulo, Brazil; ^11^ Division of Endocrinology, Indiana School of Medicine, Indianapolis, Indiana, USA; ^12^ Department of Medical Oncology and Hematology, Cantonal Hospital, Sankt Gallen, Switzerland; ^13^ International Hereditary Cancer Center, Department of Genetics and Pathology, Pomeranian Medical University, Szczecin, Poland; ^14^ Department of General Surgery; Division of Endocrinology and Metabolism Disorders; and Department of Medical Genetics, Ege University Faculty of Medicine, Izmir, Turkey; ^15^ Department of Endocrinology, King Edward Memorial (KEM) Hospital, Mumbai, India; ^16^ Nuclear Medicine and Endocrine Oncology Department, Maria Sklodowska-Curie National Research Institute of Oncology, Gliwice Branch, Gliwice, Poland; ^17^ Department of Genetic and Molecular Diagnostics of Cancer, Maria Sklodowska-Curie National Research Institute of Oncology, Gliwice Branch, Gliwice, Poland; ^18^ Department of Internal Medicine, Division of Endocrinology, Groningen University Medical Center, Groningen, The Netherlands; ^19^ Division of Endocrinology, Diabetes, Metabolism, and Nutrition, Mayo Clinic, Rochester, Minnesota, USA; ^20^ Adrenal and Hypertension Unit, Division of Endocrinology, Department of Medicine, Federal University of São Paulo, and Santa Casa Medical School, Sao Paulo, Brazil; ^21^ Department of Endocrinology, CHU de Liège, Domaine Universitaire du Sart-Tilman, Liège, Belgium; ^22^ Department of Oncologic and Urologic Surgery, The 903rd PLA Hospital, Hangzhou Medical College, Hangzhou, China; ^23^ Medicover Oldenburg MVZ, Oldenburg, Germany; ^24^ Department of Internal Medicine I, Division of Endocrinology and Diabetes, University Hospital, University of Würzburg, Würzburg, Germany; ^25^ Department of Medical Oncology, Hospital Erasto Gaertner, Curitiba, Brazil; ^26^ A.C. Camargo Cancer Center, Sao Paulo, Brazil; ^27^ Department of Therapeutic Endocrinology and Department of Surgery, Endocrinology Research Center, Moscow, Russia; ^28^ Department of Endocrinology, CETREN-UC, Faculty of Medicine, Pontificia Universidad Católica de Chile, Santiago, Chile; ^29^ Department of Endocrinology, Universitair Ziekenhuis Brussel (UZ Brussel), Vrije Universiteit Brussel (VUB), Brussels, Belgium; ^30^ Division of Endocrinology and Metabolism, Siriraj Hospital, Mahidol University, Bangkok, Thailand; ^31^ Cancer Genetics Service, Division of Medical Oncology, National Cancer Center Singapore, and Lee Kong Chian School of Medicine, Nanyang Technological University, Singapore, Singapore; ^32^ Departement of Medical Genetics, CHU de POITIERS, Poitiers, France; ^33^ Department of Medical Genetics, University of Cambridge, Cambridge Biomedical Campus, Cambridge, UK; ^34^ Clinic for Endocrine Surgery, University Clinical Center of Serbia, and School of Medicine, University of Belgrade, Belgrade, Serbia; ^35^ National Institute of Cardiology, Department of Hypertension, Warsaw, Poland; ^36^ Department of Molecular Medicine and Surgery, Karolinska Institute, and Department of Endocrinology, Karolinska University Hospital, Stockholm, Sweden; ^37^ Department of Clinical, Internal Medicine, Anesthesiology and Cardiovascular Sciences, “Sapienza” University of Rome, Rome, Italy; ^38^ Serviço de Endocrinologia, Diabetes e Metabolismo, CHULN and Faculdade de Medicina da Universidade de Lisboa, Lisbon, Portugal; ^39^ Department of Internal Medicine, Division of Endocrinology, Leiden University Medical Center, Leiden, The Netherlands; ^40^ Department of Surgery, and Department of Biomedical and Clinical Sciences, Linköping University, Linköping, Sweden; ^41^ Endocrinology in Charlottenburg, Berlin, Germany; ^42^ Department of Visceral and Thoracic Sugery, Winterthur Cantonal Hospital, Winterthur, Switzerland; ^43^ Department of Radiology, Faculty of Medicine, Albert-Ludwigs-University, Freiburg, Germany; ^44^ Department of Neuroradiology, Faculty of Medicine, Albert-Ludwigs-University, Freiburg, Germany; ^45^ Department of Nuclear Medicine, Städtisches Klinikum Karlsruhe, Karlsruhe, Germany; ^46^ Eye Center, Medical Center, Faculty of Medicine, University of Freiburg, Freiburg, Germany; ^47^ Department of Ophthalmology, University Hospital Brandenburg, Brandenburg Medical School Theodor Fontane (MHB), Brandenburg an der Havel, Germany; ^48^ Department of Gastroenterology, Faculty of Medicine, Albert-Ludwigs-University, Freiburg, Germany; ^49^ Department of Oncology-Pathology, Karolinska Institute, Stockholm, Sweden; ^50^ Department of Clinical Pathology and Cancer Diagnostics, Karolinska University Hospital, Stockholm, Sweden; ^51^ Department of Oncogenetics, A.C. Camargo Cancer Center, Sao Paulo, Brazil; ^52^ Department of Translational and Precision Medicine, “Sapienza” University of Rome, Rome, Italy; ^53^ Neuroendocrinology Laboratory, Endocrinology Institute, Almazov National Medical Research Centre, St. Petersburg, Russia; ^54^ Özel Sağlık Hospital, Centre for Endocrine Surgery, Izmir, Turkey; ^55^ Department of Internal Medicine, DIMED, University of Padua, Padua, Italy; ^56^ Department of Oncology-Pathology, Karolinska Institutet, Stockholm, Sweden; ^57^ Endocrine Section, Hospital del Salvador, Department of Medicine University of Chile, Santiago de Chile, Chile; ^58^ Department of General and Endocrine Surgery, CHU Poitiers, Poitiers, France; ^59^ Aston Medical School, Aston University, Birmingham, UK; ^60^ Department of Surgery, Kliniken Essen-Mitte, Essen, Germany; ^61^ Department II of Internal Medicine, Faculty of Medicine, and University Hospital, University of Cologne, Cologne, Germany

**Keywords:** von Hippel–Lindau disease, genotype-phenotype, tumor risk profiles, personalized preventive medicine

## Abstract

Hereditary tumor predisposition syndromes pose a challenge for early detection and timely treatment of tumors. In von Hippel–Lindau disease, desirable personalized surveillance programs are lacking due to insufficient data on genotype-specific risk profiles of individual mutations. To describe neoplastic risk profiles for carriers of pathogenic and likely pathogenic *VHL* germline mutations, our observational study recruited 1,350 participants from 40 centers worldwide. 432 different VHL germline mutations were observed, with p.Asn78Ser, p.Arg161Ter, p.Arg161Gln, p.Arg167Gln, p.Arg167Trp and p.Tyr98His being the six most frequent, occurring in a total of 493 carriers (36.5%) and in ≥30 patients each. Age-related penetrance risks for retinal hemangioblastoma, central nervous system hemangioblastoma, renal cell carcinoma, pancreatic neuroendocrine tumors and pheochromocytoma/paraganglioma in carriers of the most frequent *VHL* mutations were assessed. In addition, the number of organs affected, the frequency of surgery and the outcome are reported. Pairwise comparisons of the age-dependent tumor penetrance of these six mutations showed that 47 out of 90 pairs were significantly different. The most significant associations were found in p.Tyr98His (*n* = 19), followed by p.Arg161Ter (*n* = 10). All pairwise comparisons of mutations affecting different codons showed at least one significant (*P* < 0.05) difference, except for p.Asn78Ser vs p.Arg161Ter. Thus, tumor risk varied by *VHL* mutation type and location, but did not differ between the truncating mutation p.Arg161Ter and the missense mutation p.Asn78Ser. Our study demonstrates the importance of mutation-specific phenotype prediction. With appropriate validation, the data have important implications for risk assessment and decision making in tumor prevention for carriers of the respective VHL mutations.

## Introduction

Germline inactivation of the von Hippel–Lindau (*VHL*) tumor suppressor gene causes the autosomal-dominant inherited von Hippel–Lindau tumor disease. Affected individuals are at risk of developing various VHL-related manifestations, including retinal hemangioblastoma/angioma (RA), central nervous system hemangioblastoma (CNS-Hbl), clear cell renal cell carcinoma (ccRCC), pancreatic neuroendocrine tumors (pNETs), pheochromocytoma/paraganglioma (PPGL) and endolymphatic sac tumors (ELSTs) (Lonser *et al.* 2003). Research on VHL disease has provided important molecular data for understanding the pathogenesis of not only hereditary ccRCC, but also the much more common sporadic ccRCC. In both, sporadic and hereditary ccRCC, VHL inactivation results in the stabilization of hypoxia inducible factors, HIF-1α and HIF-2α, and activation of hypoxia-response signaling pathways that, in certain tissues, promote tumor development (Gossage *et al.* 2015, Kaelin 2022). The discovery that carcinogenesis is driven by HIF-2α has recently led to the development of a clinical HIF-2α antagonist (belzutifan) for the treatment of VHL-associated ccRCC, CNS-Hbl and pNET (Jonasch *et al.* 2021) and advanced sporadic ccRCC (Jonasch *et al.* 2024).

In VHL disease, a broad spectrum of pathogenic *VHL* mutations has been observed, ranging from single nucleotide variants to loss of the entire gene, contributing to the wide range of phenotypic manifestations of the disease. Previous studies have focused on genotype–phenotype correlations, but mainly compared mutation types (e.g., point mutations versus intraexonic deletions and/or large deletions) or regions of affected codons, limiting our knowledge of the risk profiles for individual mutations (Gallou *et al.* 2004, Maranchie *et al.* 2004, Ong *et al.* 2007, Franke *et al.* 2009, Nordstrom-O'Brien *et al.* 2010, Hong *et al.* 2019, Qiu *et al.* 2020, Chiorean *et al.* 2022). Classically, *VHL* whole-gene deletions, nonsense variants, frameshifting insertions and deletions (indels) and certain splice variants have been associated with ccRCC and Hbl (VHL type 1), while missense mutations have been associated with PPGL (type 2 VHL) (Maher *et al.* 2011). Type 2 disease can be further divided into type 2A (includes Hbl), type 2B (includes ccRCC) and type 2C disease with only PPGL. However, many variants have been reported to cause both, type 1 and type 2 disease (Tabaro *et al.* 2016) and, although rare, patients with nonsense variants have presented with type 2 disease with early-onset PPGL (Zhang *et al.* 2015). Thus, due to the limited cohort size, discrepancies in observational data and our incomplete understanding of the mechanistic effects of the variants, desirable, widely accepted, personalized surveillance and therapy plans for carriers of various *VHL* mutations are lacking, although some have been proposed (Tirosh *et al.* 2018).

With advances in technology and with longitudinal follow-up of cohorts, the opportunity arises to prospectively describe disease courses for different *VHL* mutations. As part of prognosis research (Hemingway *et al.* 2013), this knowledge provides the basis for future research and ultimately for personalized medicine. To this end, we have established a broad multicountry VHL registry with diagnostic and treatment data to establish a genotype-specific phenotype map in *VHL* germline mutation carriers.

## Materials and methods

### Study design

The VHL Risk Profile Registry is a multicenter cohort of patients with germline *VHL* mutations led by the University Hospital of Freiburg in cooperation with 39 centers worldwide and registered with the DRKS – German clinical trials registry, DRKS00032577. The study has been approved by the Ethical Committee of the Medical Faculty of the University of Freiburg and by the equivalent committees among participating centers. To be included in the registry, patients had to have a *VHL* germline mutation confirmed by molecular genetic testing.

### Participating centers

We invited colleagues of whom we knew about their dedication to clinical and/or genetic research in this field to contribute to this registry. All participating centers adhere to international guidelines for VHL (Daniels *et al.* 2023), including molecular genetic diagnosis, ophthalmoscopy including complete peripheral retinal examination, radiologic imaging with contrast-enhanced magnetic resonance imaging (MRI) of the brain and spinal cord, and MRI and/or CT of the abdomen. All centers have agreement regarding laser beam coagulation of any RA and the principles of symptoms and/or tumor sizes indicating surgical removal of Hbl of the brain, spinal cord and abdominal tumors. The search for *VHL* gene mutations was performed using an EDTA blood-derived DNA sample by Sanger sequencing for intraexonic variants and MLPA analysis for large deletions/rearrangements or by next-generation sequencing multigene panels. Only patients with molecular genetically confirmed variants class 4 and 5 (likely pathogenic and pathogenic) according to American College of Medical Genetics and Genomics (ACMG) and the Association for Molecular Pathology were accepted for participation in the registry (Richards *et al.* 2015, NGS in PPGL Study Group *et al.* 2017).

### Data

A database was set up based on a pre-defined catalog of data items including genetics, diagnosis of tumors including treatment procedures and outcome. Clinical data included age, sex and results from ophthalmoscopy, CT scans and/or MRI of the brain, spinal cord and abdomen. Treatment data included surgery details and outcome. Such investigations were established in all participating centers for VHL patients according to the international guidelines.

VHL-associated tumors included RAs, CNS-Hbls, ccRCCs, pNETs, PPGLs and ELSTs (Lonser *et al.* 2003). All tumors were confirmed by state of the art imaging and, if removed, additionally by histopathology. All data were collected until November 2023, with a second systematic review of all participating centers until January 1, 2024. All data have been independently checked by at least six colleagues of the Freiburg team and at least four colleagues from the Sao Paulo team. Patients i) without clinical data (*n* = 20) and ii) without molecular screening analysis of the *VHL* gene (*n* = 23) were excluded from the analysis, resulting in a total of 1,350 participants. In addition to tumor manifestations, outcomes of interests were: the occurrence of metastases of ccRCC, pNET or PPGL, loss of function of one or both eyes and/or ears, permanent severe CNS deficits, kidney failure, steroid dependency and postoperative endocrine or exocrine pancreas dysfunction.

### Statistical analysis

Data from participants were described using the mean (SD) for continuous variables and frequency (proportion) for categorical variables.

For mutations observed in at least 30 participants, we performed detailed analyses in order to define relevant mutation-specific differences (Oakes 2001). This included estimation of age-related penetrance using Kaplan–Meier estimator, the calculation of number of affected (bilateral) organs and of treatment procedures, and a description of outcome data.

In Kaplan–Meier analysis, participants without respective manifestations were censored at the age of their last visit. Log-rank test was used for pairwise comparisons of age-related penetrance curves for different *VHL* variants and Cox regression to estimate the instantaneous risk for the manifestation (hazard ratio; HR).

For comparison of distributions of various characteristics across *VHL* variants, a Χ^2^-test for binary variables or Kruskal–Wallis rank sum test for count variables was conducted.

*P* values less than 0.05 were considered nominally statistically significant. To correct for multiple testing, *P* values of log-rank test were adjusted using the Benjamini–Hochberg approach (Benjamini & Hochberg 1995). All analyses were conducted using the GraphPad Prism 9.3.1 or using the R software (https://www.r-project.org/).

## Results

### Study population

The International VHL Risk Profile Registry (as of April 1, 2024) contained data from 1,350 patients with confirmed germline class 4 or 5 variants of the *VHL* gene and who had clinical data available to perform deep phenotyping for VHL syndrome. Worldwide, 40 centers in 21 countries contributed to the registry (Table 1 and Supplementary Table 1 (see section on Supplementary materials given at the end of the article)). Age distribution at final observation of the participants was 40.2 (±16.7) years. There were 704 (52.1%) females. 107 participants died by age 50.6 (±16.2) years. In 90.3% of the deceased for whom the cause of death was known (*n* = 72), VHL was listed as its cause. Detailed data based on ophthalmoscopy, CT and/or MRI of the CNS and the abdomen were available for PPGL in 1,338 (99.1%), ccRCC in 1,333 (98.7%), pNET in 1,333 (98.7%), CNS-Hbl in 1,322 (97.9%) and RA in 1,315 (97.4%) of the participants.

**Table 1:** Characteristics of 1,350 participants with germline *VHL* mutations (ACMG variants class 4 or 5).

Characteristics		
	Frequency (number, %)	Age in years at last visit (mean, SD)
Overall	1,350 (100)	40.3 (±16.7)
Female sex	704 (52.1)	42.1 (±16.3)
Geographic region		
Asia (5 centers)*	98 (7.3)	35.2 (±12.7)
Europe (23 centers)^†^	1,009 (74.7)	42.4 (±16.6)
North America (3 centers)	60 (4.4)	29.3 (±17.5)
South America (9 centers)^‡^	183 (13.6)	34.7 (±16.0)
*VHL* mutations		
Missense mutations	907 (67.2)	40.7 (±17.3)
Truncations/deletions (including frame shift mutations and splice site mutations)	443 (32.8)	39.3 (±15.4)

Disease manifestations			
	Frequency (number, %)	Age in years at first diagnosis (mean, SD)	Age ≥65 years at first diagnosis (number, %)
Retinal hemangioblastoma	654 (48.4)	34.2 (±15.7)	25 (3.8)
Intracranial haemangioblastoma	618 (45.8)	33.0 (±13.5)	18 (2.9)
Spinal hemangioblastoma	608 (45.0)	36.0 (±13.9)	18 (3.0)
Renal cell carcinoma	399 (29.6)	37.5 (±12.0)	7 (1.8)
Pancreatic neuroendocrine tumor	248 (18.4)	39.8 (±13.7)	11 (4.4)
Pheochromocytoma/paraganglioma	527 (39.0)	28.4 (±16.1)	13 (2.5)
Endolymphatic sac tumor	47 (3.5)	32.5 (±13.4)	0

* Turkey, China, India, Singapore, Thailand.

^†^ Belgium, France, Germany, UK, Italy, Netherlands, Poland, Portugal, Russia, Serbia, Sweden, Switzerland.

^‡^ Argentina, Brazil, Chile.

### Germline VHL variants

196 different germline mutations of the *VHL* gene have been found in the 1,350 participants (Supplementary Table 2). In addition, 236 participants had large deletions/rearrangements of 1–3 exons; these are each counted separately, since a previous analysis showed different breakpoints (Franke *et al.* 2009). Pathogenic (class 5) variants were found in 1,202 participants, whereas 148 participants carry variants classified as likely pathogenic (class 4). Missense mutations were present in 907, truncating mutations in 443 participants.

The six most frequent distinct mutations are c.233A>G (p.Asn78Ser), present in 30 participants, c.481C>T (p.Arg161Ter), present in 42, c.482G>A (p.Arg161Gln), present in 43, c.500G>A (p.Arg167Gln) present in 68, c.499C>T (p.Arg167Trp), present in 84, and c.292T>C (p.Tyr98His), present in 226 participants. All these six are pathogenic variants (class 5). In all, 493 participants carrying the six most frequent mutations comprise 36.5% in this new international VHL registry. The p.Tyr98His mutation is endemic in the Black Forest, South Germany; such participants were contributed by five centers only. Participants of the other five mutations have been contributed by 9–21 different centers (Table 2); the maximum of contributed participants per center for these five mutations was eight (p.Arg167Gln) to 25 (p.Arg167Trp).

**Table 2:** Clinical characteristics of the six most frequent *VHL* mutations.

		p.Asn78Ser	p.Arg161Ter	p.Arg161Gln	p.Arg167Gln	p.Arg167Trp	p.Tyr98His
Registrants (*n*)		30	42	43	68	84	226
Contributing centers (*n*)		9	12	10	21	18	5
Age at last check-up	Mean	42.0	35.7	32.0	39.6	36.8	48.0
	SD	15.1	14.1	15.1	14.8	15.9	19.2
Involved organs/organ systems (%)	0	3.3	14.3	4.7	8.8	15.5	19.9
	1	13.3	11.9	46.5	11.8	27.4	27.4
	2	33.3	28.6	18.6	36.8	22.6	36.7
	3	40.0	35.7	18.6	20.6	14.3	14.6
	4	6.7	9.5	11.6	17.6	14.3	1.3
	5	3.3	0.0	0.0	4.4	6.0	0.0
	Mean	2.4	2.1	1.9	2.4	2.0	1.5
	SD	1.0	1.2	1.1	1.3	1.5	1.0
Bilaterally involved organs (*n*, %)		14 (46.7)	14 (33.3)	18 (41.9)	30 (44.1)	27 (32.1)	40 (17.7)
Operations per registrant (%)	0	23.3	38.1	7.0	23.5	25.0	43.8
	1	10.0	9.5	41.9	23.5	23.8	34.1
	2	30.0	19.0	23.3	22.1	26.2	13.7
	3	13.3	11.9	18.6	8.8	9.5	5.3
	4	3.3	4.8	2.3	8.8	4.8	2.2
	5	6.7	4.8	7.0	7.4	1.2	0.0
	6	3.3	2.4	0.0	1.5	2.4	0.4
	7	3.3	0.0	0.0	0.0	2.4	0.0
	8	3.3	7.1	0.0	4.4	1.2	0.4
	9	0.0	0.0	0.0	0.0	1.2	0.0
	10	3.3	0.0	0.0	0.0	1.2	0.0
	11	0.0	2.4	0.0	0.0	1.2	0.0
	Mean	2.6	2.2	1.9	2.1	2.1	0.9
	SD	2.5	2.7	1.3	2.0	2.3	1.2
Outcome: lost organ function (*n*, %)		13 (43.3)	16 (38.1)	14 (32.6)	30 (44.1)	26 (31.0)	40 (17.7)

Of the less frequent mutations, 164 are present in 1–5 participants, 18 in 6–10 participants, seven in 11–20 participants and one in 22 participants (Supplementary Table 2).

### Clinical manifestations and interventions

Of the 1,350 participants, 654 (48.4%) had RAs, among whom 205 (31.3%) had bilateral involvement. CNS-Hbls were found in 815/1,350 participants (60.4%); of these, 618 (75.8%) had intracranial Hbls and 608 (74.6%) had spinal Hbls. ccRCCs were found in 399/1,350 (29.6%) participants, with 221 (55.4%) having bilateral tumors. pNETs were noted in 248/1,350 (18.4%), PPGLs in 527/1,350 (39.0%) and ELSTs in 47/1,350 (3.5%) participants (Table 1). The high percentage of PPGL may be explained by the high number of p.Tyr98His carriers in our international VHL registry.

Unilateral vision impairment occurred in 186/654 (28.4%) participants with RAs, and bilateral retinal involvement with more than 50% vision reduction/complete blindness was identified in 32/654 (4.9%) participants. Enucleation of one or both eyes was performed in 36/654 (5.5%) participants. In participants with CNS-Hbl, permanent severe neurological deficits occurred in 168/815 (20.6%) participants. Metastases were present in 42/399 (10.5%) participants with ccRCC, in 17/248 (6.9%) participants with pNET and in 11/527 (2.1%) participants with PPGL.

CNS-Hbl surgeries were performed in 556/815 (68.2%) participants, with 279 (50.2%) having more than one operation. Renal surgery including ablative treatment (thermoablation/cryotherapy) was performed in 292/399 (73.2%) participants with ccRCC; bilateral procedures were performed in 140 of these 292 participants (47.9%). Of the 292 participants undergoing ccRCC treatment, 90 (30.8%) had tumor recurrence in the same kidney requiring re-intervention. Among the 140 participants who required intervention for bilateral ccRCC, 16 (11.4%) experienced endstage renal failure and required dialysis with subsequent kidney transplantation in three participants. Of the 248 participants with pNETs, 89 (35.9%) had tumor enucleation or Whipple operation. Of the 527 participants with PPGL, 153 (29.0%) had two or more operations and 36 (6.8%) became steroid dependent. ELSTs were found in 47 participants, 42/47 (89.4%) had unilateral and 5/47 (10.6%) had bilateral hearing loss, three received a cochlear implant. 3.8% of RAs, 2.9% of intracranial Hbls, 3.0% of spinal Hbls, 1.8% of ccRCCs, 4.4% of pNETs and 2.5% of PPGLs were diagnosed at age 65 years or older.

### Risk profiles in participants with one of the six most frequent VHL mutations

**Figure 1:**
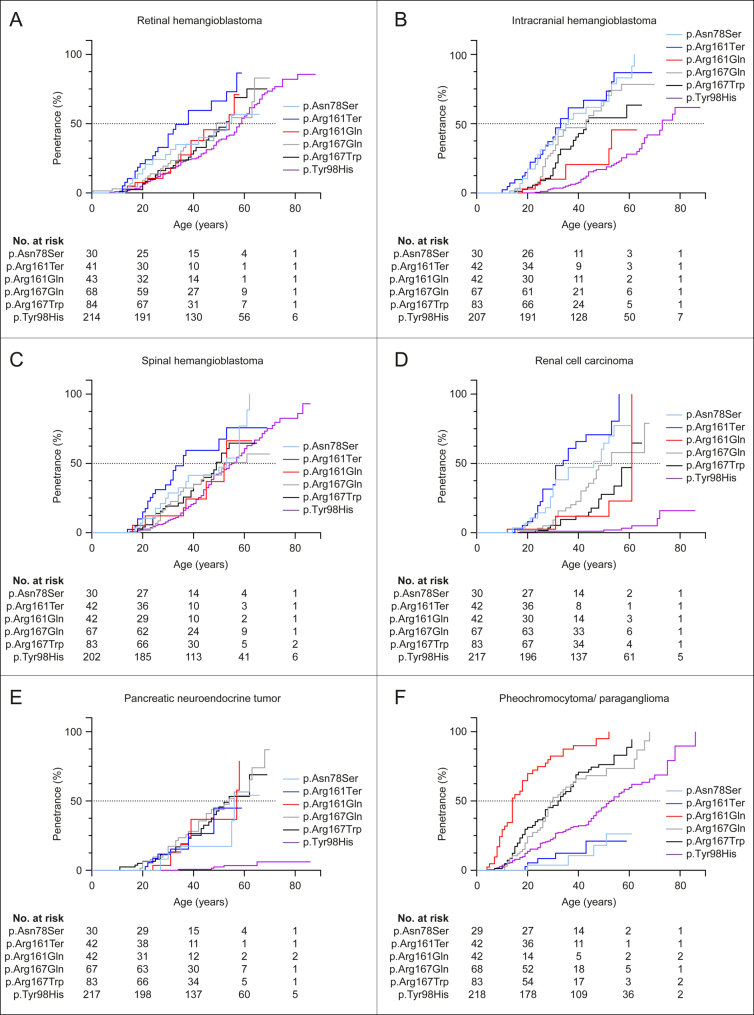
Penetrance curves for each of the six most frequent *VHL* mutations per tumor manifestation. Penetrance for age at diagnosis was estimated using the Kaplan–Meier estimator. The curves partially show distinct courses which are statistically significant (Supplementary Table 3).

We analyzed in detail clinical data of the 493 participants with the six most frequent mutations: p.Asn78Ser, p.Arg161Ter, p.Arg161Gln, p.Arg167Gln, p.Arg167Trp and p.Tyr98His (Fig. 1, Table 2). The following clinical parameters were chosen to phenotypically characterize mutations: i) penetrance; ii) number of involved organs/organ systems; iii) bilateral tumors in paired organs; iv) number of treatment procedures per patient; and v) outcome, for which we summarized eyes and ears without function, presence of metastases, hemodialysis/transplantation, postoperative pancreatic insufficiency, steroid-dependency and/or permanent severe CNS deficits. Pairwise comparisons for each of the six mutations resulted in 15 pairs for each parameter.

#### Penetrance

We observed highly significant differences in age-related penetrance (Fig. 1). Pairwise comparison of the penetrance curves across the six tumor manifestations (6 × 15 = 90 pairs) revealed 47 pairs to be nominally significantly (*P* < 0.05) different, of which 43 remained significant after multiple testing correction (*P*_adjust_ < 0.05), including 29 pairs with *P* < 0.0001 (Supplementary Table 3). All pairwise comparisons of mutations showed at least one significant (*P* < 0.05) difference, except for p.Asn78Ser versus p.Arg161Ter and p.Arg167Trp versus p.Arg167Gln, as reflected by their respective similar penetrance curves (Fig. 1). Most significant associations with *P* < 0.0001 were found in p.Tyr98His (*n* = 19), followed by p.Arg161Ter (*n* = 10). Compared to patients carrying the p.Tyr98His mutation, carriers of other mutations were at higher risk for many of the VHL-associated tumors. For instance, we observed that p.Arg161Ter carriers had an 81-fold higher risk for ccRCC (HR = 81, 95% confidence interval (CI) 30–221). This result, however, should be interpreted with caution due to sparseness of data (Greenland *et al.* 2016). Only for PPGL, the risk in p.Arg161Ter (HR 0.4, 95% CI 0.1–0.9) and p.Asn78Ser (HR 0.3, 95% CI 0.1–0.9) carriers was reduced in comparison to p.Tyr98His carriers.

#### Number of the involved organs

The number of organs involved (Table 2) per registrant (range: 0–5) varied significantly among the six most frequent mutations (*P* = 1.0E-07), with the highest number in those with the p.Asn78Ser and p.Arg161Gln mutations (mean 2.4) and the lowest number in those with the p.Tyr98His mutation (mean 1.5).

#### Bilaterally involved organs

Bilateral tumors in paired organs occurred in 143 of the 493 most frequent mutations carriers (29.0%) with significantly varying proportions (*P* = 1.3E-05). 47% of p.Asn78Ser carriers had bilateral organ involvement compared to only 18% of p.Tyr98His carriers (Table 2).

#### Number of operations

Total number of operations/treatment procedures per registrant (range 0–11) revealed also significant differences among the participants with the six most frequent mutations (*P* = 4.1E-10; Table 2). p.Asn78Ser carriers had the most frequent procedures, with mean 2.6 procedures per patient, compared to the lowest in those with p.Tyr98His, with mean 0.9 procedures per patient.

#### Outcome

Outcome scoring for permanent impairments including blindness of one or both eyes, persistent severe neurological defects, need of dialysis, metastases due to ccRCC, pNETs or PPGL, postoperative pancreatic insufficiency, steroid dependency and deafness of one or both ears revealed statistically significant differences among carriers of the six most frequent mutations (*P* = 5.8E-5). The worst outcome was again observed in participants with p.Asn78Ser (43% of participants), and the best in those with p.Tyr98His (18%, Table 2).

## Discussion

By this newly established multicenter, multinational registry comprising 1,350 individuals with *VHL* germline mutations, we studied mutation-specific risk profiles with respect to different tumor manifestations, organs involved, performed treatments and outcomes. We addressed this issue by analyzing clinical phenotypes and outcomes in carriers of the six most frequent *VHL* mutations present in a third of our participants and found, by collaboration of 40 centers, significant differences in many of the analyzed aspects. All previous studies on VHL genotype–phenotype correlations have mainly focused on age-related penetrance; in contrast, and for the first time, we also evaluated the number of affected organs, treatment procedures and outcomes.

Although not influencing clinical decision making, VHL is still classified as type 1 or 2 disease based on the frequency of ccRCC and PPGL (Nielsen *et al.* 2016). Patients with truncating mutations or exon deletions tend to develop a type 1 phenotype, whereas type 2 VHL is characterized by missense mutations, an observation supported by several publications, including recent ones (Tamura *et al.* 2023). Our study has shown that such generalizations must be considered with caution. Although mainly missense substitutions at the surface of pVHL are thought to cause type 2 disease (predominantly PPGL) (Stebbins *et al.* 1999, Ong *et al.* 2007), we were still surprised by the distinct type 1 phenotype (ccRCC and Hbl) of the missense mutation p.Asn78Ser (a ‘deep’ missense mutation located at the protein core) (Ong *et al.* 2007) contributed by nine centers from eight countries. Remarkably, missense mutations affecting the highly conserved amino acids 74–90 (Woodward *et al.* 2000) and including surface missense mutations have previously been associated with ccRCC risk (Gallou *et al.* 2004). Notably, p.Arg167Gln (a surface missense mutation) had a similar risk of intracranial Hbl to p.Asn78Ser. In addition, even after adjustment for multiple comparisons, p.Arg161Gln had a significantly higher PPGL penetrance compared to p.Arg167Trp and p.Arg167Gln, suggesting that even among surface missense variants phenotypes are different. Moreover, the variants p.Arg64Pro, p.Val84Leu, p.Phe119Leu and p.Leu188Val are classically considered to be VHL type 2C variants (PPGL only) (Clifford *et al.* 2001, Hoffman *et al.* 2001). However, we found VHL manifestations other than PPGL in at least one carrier of all these amino acid substitutions, raising the question of the existence of an isolated type 2C phenotype that would allow omitting surveillance for other manifestations (Supplementary Table 4). In VHL disease, correlations of distinct specific mutations are limited to those which have been identified with founder effects (Green *et al.* 1986, Lamiell *et al.* 1989, Brauch *et al.* 1995). A well-characterized mutation is the p.Tyr98His mutation (Brauch *et al.* 1995), which is endemic in the Black Forest and was described as a type 2A mutation, with PPGL and CNS-Hbl as the typical manifestations (Brauch *et al.* 1995, Nielsen *et al.* 2016, Maher & Sandford 2019). However, we show that carriers of p.Tyr98His have a lower risk of developing PPGL and intracranial Hbl than those with missense mutations affecting amino acids 161 and 167. Our analyses suggest that different *VHL* variants have different organ-specific cellular and molecular functions that explain their different tumor propensity. The best known function of pVHL is as a ubiquitin ligase for HIF transcription factors (Gossage *et al.* 2015). *VHL* alleles have been shown to differ in the extent of HIF downregulation, with relative HIF levels highest in mutations causing type 1 disease and lower in type 2 disease (Clifford *et al.* 2001, Hoffman *et al.* 2001, Kaelin 2022). However, although p.Arg161Gln and p.Arg167Gln show no significant difference in penetrance curves for ccRCC, RA, pNET and spinal Hbl, their risk for intracranial Hbl and PPGL differs. All five analyzed missense mutations show, after correction for multiple testing, overlapping penetrance curves for RA and spinal Hbl. For pNET and ccRCC, p.Tyr98His has a significantly lower risk, whereas for PPGL, p.Asn78ser has the lowest risk. These observations implicate organ-specific involvement in unknown subcellular and molecular functions of these variants beyond HIF degradation, which require further investigation. It would be instructive to carry out similar analyses for all *VHL* mutations. However, since most *VHL* variants are rare, it is doubtful that enough patients will ever be sequenced to reliably predict neoplasia risk for each possible *VHL* variant. As experimental approaches for characterizing variant effects improve (Findlay 2021), large genotype–phenotype maps, such as ours, will become increasingly important for correlating and validating experimental findings. This combination of approaches may, in the future, lead to the development of mutation-specific tumor surveillance and therapy that is highly sought after by patients.

## Limitations

A limitation of our study is the retrospective nature of data collection. Even with a worldwide collaboration of 40 centers, however, sample size and clinical information (e.g., missing age of diagnosis) are limited. More detailed analysis was thus not possible.

## Conclusions

Our results, derived from the analysis of the largest available cohort with *VHL* germline mutations (to our knowledge), reveal previously unknown mutation- and organ-specific differences in tumor penetrance, number of organs affected, number of surgeries and outcome. Further research, e.g., the correlation of large genotype–phenotype maps with experimental research, may be needed to obtain reliable neoplasia risk estimates for carriers of different *VHL* mutations. Our study emphasizes the importance of mutation-specific tumor prediction. These data have important implications for risk assessment and decision making in tumor prevention for carriers of the respective *VHL* mutations.

## Supplementary materials



## Declaration of interest

Joanne Ngeow is an Associate Editor of *Endocrine-Related Cancer*. Joanne Ngeow was not involved in the review or editorial process for this paper, on which she is listed as an author. The authors declare that they have no known competing interests that could influence the work reported in this paper.

## Funding

The study was supported in part by grants from the European Union (grant LSHC-CT-2005-518200) and the German Cancer Foundation (grant 107995) to HPHN; by the Deutsche Forschungsgemeinschafthttps://doi.org/10.13039/501100001659 (DFG, German Research Foundation) – Project-ID 431984000-SFB 1453 to AG, PS, AK and ENH; by the Sao Paulo Research Foundationhttps://doi.org/10.13039/501100001807 (FAPESP) grant 2019/15873-6 to MQA; in addition, by the Fondecythttps://doi.org/10.13039/501100002850 grant #1190419 to RB and TU; and ANID ANILLO ACT210039 to RB. ERM acknowledges funding from NIHR Cambridge Biomedical Research Centrehttps://doi.org/10.13039/501100018956 (NIHR203312) and VHL UK/Ireland.

## Author contribution statement

HPHN, AG and PS conceptualized the analysis. AG and PS calculated descriptive statistics and comparisons and created the penetrance graphs and tables. MQA, LSS, FFC and FS performed the classification of the different variants. MQA, LSS, FFC and FS accessed and verified data analysis and statistical comparisons. AK, ENH, AG, PS, MQA, RB and TU contributed to funding acquisition. HPHN, AG and PS wrote the initial draft. ENH, AK, MQA, CE and ERM critically reviewed and edited the original draft. All authors contributed to data acquisition and collection, data curation, data interpretation, literature research and revision of the manuscript. All authors had final responsibility for the decision to submit for publication.

## References

[bib2] Benjamini Y & Hochberg Y 1995 Controlling the false discovery rate – a practical and powerful approach to multiple testing. J Roy Stat Soc B Stat Methodol 57 289–300. (10.1111/j.2517-6161.1995.tb02031.x)

[bib3] Brauch H, Kishida T, Glavac D, et al. 1995 Von Hippel-Lindau (VHL) disease with pheochromocytoma in the Black Forest region of Germany: evidence for a founder effect. Hum Genet 95 551–556. (10.1007/bf00223868) 7759077

[bib4] Chiorean A, Farncombe KM, Delong S, et al. 2022 Large scale genotype- and phenotype-driven machine learning in von Hippel-Lindau disease. Hum Mutat 43 1268–1285. (10.1002/humu.24392) 35475554 PMC9356987

[bib5] Clifford SC, Cockman ME, Smallwood AC, et al. 2001 Contrasting effects on HIF-1alpha regulation by disease-causing pVHL mutations correlate with patterns of tumourigenesis in von Hippel-Lindau disease. Hum Mol Genet 10 1029–1038. (10.1093/hmg/10.10.1029) 11331613

[bib6] Daniels AB, Tirosh A, Huntoon K, et al. 2023 Guidelines for surveillance of patients with von Hippel-Lindau disease: consensus statement of the international VHL surveillance guidelines consortium and VHL alliance. Cancer 129 2927–2940. (10.1002/cncr.34896) 37337409

[bib7] Findlay GM 2021 Linking genome variants to disease: scalable approaches to test the functional impact of human mutations. Hum Mol Genet 30 R187–R197. (10.1093/hmg/ddab219) 34338757 PMC8490018

[bib8] Franke G, Bausch B, Hoffmann MM, et al. 2009 Alu-Alu recombination underlies the vast majority of large VHL germline deletions: molecular characterization and genotype-phenotype correlations in VHL patients. Hum Mutat 30 776–786. (10.1002/humu.20948) 19280651

[bib9] Gallou C, Chauveau D, Richard S, et al. 2004 Genotype-phenotype correlation in von Hippel-Lindau families with renal lesions. Hum Mutat 24 215–224. (10.1002/humu.20082) 15300849

[bib10] Gossage L, Eisen T & Maher ER 2015 VHL, the story of a tumour suppressor gene. Nat Rev Cancer 15 55–64. (10.1038/nrc3844) 25533676

[bib11] Green JS, Bowmer MI & Johnson GJ 1986 Von Hippel-Lindau disease in a Newfoundland kindred. CMAJ 134 133–138. 3942913 PMC1490656

[bib12] Greenland S, Mansournia MA & Altman DG 2016 Sparse data bias: a problem hiding in plain sight. BMJ 352 i1981. (10.1136/bmj.i1981) 27121591

[bib13] Hemingway H, Croft P, Perel P, et al. 2013 Prognosis research strategy (PROGRESS) 1: a framework for researching clinical outcomes. BMJ 346 e5595. (10.1136/bmj.e5595) 23386360 PMC3565687

[bib14] Hoffman MA, Ohh M, Yang H, et al. 2001 von Hippel-Lindau protein mutants linked to type 2C VHL disease preserve the ability to downregulate HIF. Hum Mol Genet 10 1019–1027. (10.1093/hmg/10.10.1019) 11331612

[bib15] Hong B, Ma K, Zhou J, et al. 2019 Frequent mutations of VHL gene and the clinical phenotypes in the largest Chinese cohort with von Hippel-Lindau disease. Front Genet 10 867. (10.3389/fgene.2019.00867) 31620170 PMC6759728

[bib16] Jonasch E, Donskov F, Iliopoulos O, et al. 2021 Belzutifan for renal cell carcinoma in von Hippel-Lindau disease. N Engl J Med 385 2036–2046. (10.1056/nejmoa2103425) 34818478 PMC9275515

[bib17] Jonasch E, Bauer TM, Papadopoulos KP, et al. 2024 Phase I LITESPARK-001 study of belzutifan for advanced solid tumors: extended 41-month follow-up in the clear cell renal cell carcinoma cohort. Eur J Cancer 196 113434. (10.1016/j.ejca.2023.113434) 38008031

[bib18] Kaelin WG Jr 2022 Von Hippel-Lindau disease: insights into oxygen sensing, protein degradation, and cancer. J Clin Invest 132 e162480. (10.1172/jci162480) 36106637 PMC9479583

[bib19] Lamiell JM, Salazar FG & Hsia YE 1989 von Hippel-Lindau disease affecting 43 members of a single kindred. Medicine 68 1–29. (10.1097/00005792-198901000-00001) 2642584

[bib20] Lonser RR, Glenn GM, Walther M, et al. 2003 von Hippel-Lindau disease. Lancet 361 2059–2067. (10.1016/S0140-6736(03)13643-4) 12814730

[bib22] Maher ER & Sandford RN 2019 von Hippel-Lindau disease: an update. Curr Genet Med Rep 7 227–235. (10.1007/s40142-019-00180-9)

[bib21] Maher ER, Neumann HP & Richard S 2011 von Hippel-Lindau disease: a clinical and scientific review. Eur J Hum Genet 19 617–623. (10.1038/ejhg.2010.175) 21386872 PMC3110036

[bib23] Maranchie JK, Afonso A, Albert PS, et al. 2004 Solid renal tumor severity in von Hippel Lindau disease is related to germline deletion length and location. Hum Mutat 23 40–46. (10.1002/humu.10302) 14695531

[bib24] NGS in PPGL (NGSnPPGL) Study Group, Toledo RA, Burnichon N, Cascon A, et al. 2017 Consensus Statement on next-generation-sequencing-based diagnostic testing of hereditary phaeochromocytomas and paragangliomas. Nat Rev Endocrinol 13 233–247. (10.1038/nrendo.2016.185) 27857127

[bib25] Nielsen SM, Rhodes L, Blanco I, et al. 2016 Von Hippel-Lindau disease: genetics and role of genetic counseling in a multiple neoplasia syndrome. J Clin Oncol 34 2172–2181. (10.1200/jco.2015.65.6140) 27114602

[bib26] Nordstrom-O'Brien M, van der Luijt RB, van Rooijen E, et al. 2010 Genetic analysis of von Hippel-Lindau disease. Hum Mutat 31 521–537. (10.1002/humu.21219) 20151405

[bib27] Oakes D 2001 Biometrika centenary: survival analysis. Biometrika 88 99–142. (10.1093/biomet/88.1.99)

[bib28] Ong KR, Woodward ER, Killick P, et al. 2007 Genotype-phenotype correlations in von Hippel-Lindau disease. Hum Mutat 28 143–149. (10.1002/humu.20385) 17024664

[bib29] Qiu J, Zhang K, Ma K, et al. 2020 The genotype-phenotype association of von hipple Lindau disease based on mutation locations: a retrospective study of 577 cases in a Chinese population. Front Genet 11 532588. (10.3389/fgene.2020.532588) 33362845 PMC7762453

[bib30] Richards S, Aziz N, Bale S, et al. 2015 Standards and guidelines for the interpretation of sequence variants: a joint consensus recommendation of the American College of Medical Genetics and Genomics and the Association for Molecular Pathology. Genet Med 17 405–424. (10.1038/gim.2015.30) 25741868 PMC4544753

[bib31] Stebbins CE, Kaelin WG Jr & Pavletich NP 1999 Structure of the VHL-ElonginC-ElonginB complex: implications for VHL tumor suppressor function. Science 284 455–461. (10.1126/science.284.5413.455) 10205047

[bib32] Tabaro F, Minervini G, Sundus F, et al. 2016 VHLdb: a database of von Hippel-Lindau protein interactors and mutations. Sci Rep 6 31128. (10.1038/srep31128) 27511743 PMC4980628

[bib33] Tamura K, Kanazashi Y, Kawada C, et al. 2023 Variant spectrum of von Hippel-Lindau disease and its genomic heterogeneity in Japan. Hum Mol Genet 32 2046–2054. (10.1093/hmg/ddad039) 36905328 PMC10244221

[bib34] Tirosh A, Sadowski SM, Linehan WM, et al. 2018 Association of VHL genotype with pancreatic neuroendocrine tumor phenotype in patients with von Hippel-Lindau disease. JAMA Oncol 4 124–126. (10.1001/jamaoncol.2017.3428) 29075773 PMC5833646

[bib35] Woodward ER, Buchberger A, Clifford SC, et al. 2000 Comparative sequence analysis of the VHL tumor suppressor gene. Genomics 65 253–265. (10.1006/geno.2000.6144) 10857749

[bib36] Zhang M, Wang J, Jiang J, et al. 2015 Von Hippel-Lindau disease type 2 in a Chinese family with a VHL p.W88X truncation. Endocrine 48 83–88. (10.1007/s12020-014-0368-x) 25069792

