# Supplementary material for: Genotype-specific neoplastic risk profiles in patients with VHL disease
Source: Endocr Relat Cancer. 2025 Apr 28;32(5):e240260. doi: 10.1530/ERC-24-0260 (PMC12060576; doi:10.1530/ERC-24-0260)
Supplement: Supplementary file 1 [file supplementary_materials.pdf]

Genotype-Specific Neoplastic Risk Profiles in Patients with VHL Disease

A. Ganner *et al.*

Table of contents

**Tab**	**Title**
Table of contents	Table of contents
Supplementary Table 1	Supplementary Table 1: Contributing Countries and frequency (proportion) of participants.
Supplementary Table 2	Supplementary Table 2: List of *VHL* germline mutations observed in the participants.
Supplementary Table 3	Supplementary Table 3: Results of pairwise comparisons of age-related penetrance curves and of Cox regression per manifestation.
Supplementary Table 4	Supplementary Table 4: List of VHL germline variants with PPGL only (type 2C) phenotype

Supplementary Table 1: Contributing Countries and frequency (proportion) of participants.

Country	Frequency	Proportion (%)
Germany	578	42,8
Italy	162	12
Poland	131	9,7
Argentina	97	7,2
Brazil	71	5,3
USA	60	4,4
Belgium	31	2,3
Switzerland	30	2,2
India	30	2,2
Turkey	29	2,1
Netherlands	25	1,9
Russia	17	1,3
China	16	1,2
Chile	15	1,1
Singapore	12	0,9
Thailand	11	0,8
France	11	0,8
UK	9	0,7
Serbia	6	0,4
Sweden	5	0,4
Portugal	4	0,3
**Total**	**1350**	**100**

Supplementary Table 2: List of *VHL* germline mutations observed in the participants.

VHL mutation	Frequency carriers	Frequency contributing centers	Codon	VHL protein variant	ACMG class
**c.292T>C**	**226**	**5**	**98**	**p.Tyr98His**	**5**
**c.499C>T**	**84**	**18**	**167**	**p.Arg167Trp**	**5**
**c.500G>A**	**68**	**21**	**167**	**p.Arg167Gln**	**5**
**c.482G>A**	**43**	**10**	**161**	**p.Arg161Gln**	**5**
**c.481C>T**	**42**	**12**	**161**	**p.Arg161Ter**	**5**
**c.233A>G**	**30**	**9**	**78**	**p.Asn78Ser**	**5**
c.227-229delTCT	22	6	76	p.Phe76del	5
c.257C>T	16	3	86	p.Pro86Leu	5
c.486C>G	15	5	162	p.Cys162Trp	5
c.277G>A	13	4	93	p.Gly93Ser	5
c.467A>G	13	4	156	p.Tyr156Cys	5
c.562C>G	12	3	188	p.Leu188Val	5
c.575C>T	12	1	192	p.Pro192Leu	4
c.599G>C	11	1	200	p.Arg200Pro	4
c.191G>C	10	5	64	p.Arg64Pro	5
c.194C>T	10	4	65	p.Ser65Leu	5
c.256C>G	10	3	86	p.Pro86Ala	5
c.407T>C	10	6	136	p.Phe136Ser	5
c.481C>G	10	3	161	p.Arg161Gly	5
c.266T>C	10	1	89	p.Leu89Pro	5
c.256C>T	9	4	86	p.Pro86Ser	5
c.320G>A	9	1	107	p.Arg107His	5
c.548C>A	9	2	183	p.Ser183Ter	5
c.464-1G>A	9	3	NA	p.?	5
c.340G>A	8	1	114	p.Gly114Ser	5
c.496G>T	8	5	166	p.Val166Phe	5
c.593T>A	8	1	198	p.Leu198Gln	5
c.193T>C	7	2	65	p.Ser65Pro	5
c.194C>A	7	3	65	p.Ser65Ter	5
c.463+2T>G	7	3	NA	p.?	5
c.239G>T	6	3	80	p.Ser80Ile	5
c.340+617C>G(382C>G)	6	1	NA	p.?	4
c.203C>A	5	1	68	p.Ser68*	5
c.217C>T	5	2	73	p.Gln73Ter	5
c.241C>T	5	3	81	p.Pro81Ser	4
c.244C>G	5	1	82	p.Arg82Gly	4
c.254T>C	5	2	85	p.Leu85Pro	4
c.257C>A	5	1	86	p.Pro86His	4
c.292T>A	5	1	98	p.Tyr98Asn	5
c.293A>C	5	2	98	p.Tyr98Ser	5
c.337C>T	5	2	113	p.Arg113Ter	5
c.413C>G	5	1	138	p.Pro138Arg	4
c.461C>T	5	2	154	p.Pro154Leu	5
c.463G>A	5	2	155	p.Val155Met	5
c.470C>T	5	2	157	p.Thr157Ile	5
c.593T>C	5	3	198	p.Leu198Pro	4
c.463+1G>A	5	3	NA	p.?	5
c.464-2A>G	5	1	NA	p.?	5
c.640T>G	5	2	214	p.Ter214GlyextTer14	4
c.232A>G	4	3	78	p.Asn78Asp	5
c.238A>G	4	3	80	p.Ser80Gly	5
c.250G>T	4	2	84	p.Val84Leu	5
c.263G>A	4	2	88	p.Trp88Ter	5
c.319C>G	4	3	107	p.Arg107Gly	5
c.370A>C	4	1	124	p.Thr124Pro	4
c.371C>T	4	2	124	p.Thr124Ile	5
c.388G>T	4	1	130	p.Val130Phe	5
c.397A>C	4	1	133	p.Thr133Pro	5
c.430G>T	4	1	144	p.Gly144Ter	5
c.473T>C	4	2	158	p.Leu158Pro	5
c.485G>A	4	1	162	p.Cys162Tyr	5
c.486C>A	4	2	162	p.Cys162Ter	5
c.490C>T	4	2	164	p.Gln164Ter	5
c.194C>G	3	2	65	p.Ser65Trp	5
c.202T>C	3	2	68	p.Ser68Pro	4
c.204dupG	3	2	69	p.Arg69Alafs*63	5
c.220delG	3	1	74	p.Val74Serfs*85	5
c.233A>T	3	1	78	p.Asn78Ile	5
c.262T>G	3	1	88	p.Trp88Gly	4
c.293A>G	3	2	98	p.Tyr98Cys	5
c.333C>G	3	1	111	p.Ser111Arg	5
c.335A>G	3	2	112	p.Tyr112Cys	5
c.353T>G	3	1	118	p.Leu118Arg	5
c.357C>G	3	2	119	p.Phe119Leu	5
c.374A>C	3	3	125	p.His125Pro	5
c.452T>C	3	2	151	p.Ile151Thr	5
c.472C>G	3	2	158	p.Leu158Val	5
c.478>9delAG(478_479del)	3	1	160	Frameshift	4
c.488T>A	3	2	163	p.Leu163His	5
c.488T>G	3	2	163	p.Leu163Arg	5

c.491A>T	3	1	164	p.Gln164Leu	5
c.525C>A	3	2	175	p.Tyr175Ter	5
c.533T>C	3	2	178	p.Leu178Pro	5
c.539T>G	3	1	180	p.Ile180Ser	4
c.551T>C	3	2	184	p.Leu184Pro	5
c.583C>T	3	2	195	p.Gln195Ter	5
c.340+1G>T	3	2	NA	p.?	4
c.188T>G	2	1	63	p.Leu63Arg	4
c.203C>G	2	2	68	p.Ser68Trp	4
c.208G>T	2	1	70	p.Glu70*	5
c.221T>G	2	2	74	p.Val74Gly	4
c.235C>G	2	1	79	p.Arg79Gly	4
c.238A>C	2	2	80	p.Ser80Arg	5
c.240T>G	2	1	80	p.Ser80Arg	5
c.250G>C	2	2	84	p.Val84Leu	5
c.262T>A	2	1	88	p.Trp88Arg	5
c.269A>T	2	2	90	p.Asn90Ile	5
c.277G>C	2	1	93	p.Gly93Arg	5
c.277G>T	2	1	93	p.Gly93Cys	5
c.294C>G	2	2	98	p.Tyr98Ter	5
c.311G>T	2	1	104	p.Gly104Val	4
c.319C>A	2	1	107	p.Arg107Ser	4
c.344A>G	2	1	115	p.His115Arg	5
c.353T>C	2	2	118	p.Leu118Pro	5
c.383T>C	2	1	128	p.Leu128Pro	5
c.388G>A	2	1	130	p.Val130Ile	4
c.389T>G	2	1	130	p.Val130Gly	5
c.392A>C	2	1	131	p.Asn131Thr	5
c.394C>T	2	1	132	p.Gln132Ter	5
c.404T>A	2	1	135	p.Leu135Ter	5
c.407T>G	2	1	136	p.Phe136Cys	5
c.439A>G	2	1	147	p.Ile147Val	4
c.445G>C	2	1	149	p.Ala149Pro	5
c.445_458del14bp	2	1	150	p.Asn150SerfsTer19	4
c.482G>C	2	1	161	p.Arg161Pro	4
c.533T>A	2	1	178	p.Leu178Gln	5
c.541delG	2	1	181	p.Val181Serfs*21	5
c.552delC	2	1	185	p.Tyr185Thrfs*17	4
c.563T>G	2	1	188	p.Leu188Arg	4
c.180insC	1	1	62	p.Val62Argfs*70	5
c.217dupC	1	1	73	p.Gln73Profs*59	5
c.219_232delGGTCATCTTCTGCA	1	1	74	p.Val74Serfs*53	5
c.236_241delGCAGTC	1	1	79	p.Arg79_Ser80del	4
c.242C>A	1	1	81	p.Pro81Gln	5
c.245G>T	1	1	82	p.Arg82Leu	5
c.264G>T	1	1	88	p.Trp88Cys	5
c.269delA	1	1	90	p.Asn90Thrfs*69	5
c.283C>G	1	1	95	p.Pro95Ala	4
c.292T>G	1	1	98	p.Tyr98Asp	4
c.296_308del	1	1	99	p.Pro99Leufs*56	5
c.295-305dup	1	1	103	p.Pro103Glnfs*60	4
c.308C>A	1	1	103	p.Pro103His	4
c.309_322del14	1	1	104	p.Gly104Hisfs*23	5
c.316insGCC	1	1	106	p.Arg108dup	4
c.314dup	1	1	107	p.Arg107Profs*25	5
c.320G>C	1	1	107	p.Arg107Pro	5
c.334T>C	1	1	112	p.Tyr112His	5
c.336C>G	1	1	112	p.Tyr112Ter	5
c.339delA	1	1	114	p.Gly114Valfs*45	5
c.344A>T	1	1	115	p.His115Leu	4
c.351G>C	1	1	117	p.Trp117Cys	5
c.352C>G	1	1	118	p.Leu118Val	4
c.358A>G	1	1	120	p.Arg120Gly	5
c.360A>C	1	1	120	p.Arg120Ser	4
c.361delG	1	1	121	p.Asp121Metfs*38	5
c.362A>G	1	1	121	p.Asp121Gly	5
c.369delThr124HisfsTer35	1	1	124	p.Thr124Hisfs*35	5
c.374dup	1	1	125	p.His125Glnfs*7	5
c.382C>T	1	1	128	p.Leu128Phe	5
c.383T>G	1	1	128	p.Leu128Arg	5
c.386T>C	1	1	129	p.Leu129Pro	5
c.343_395dup	1	1	132	p.Gln132Hisfs*45	5
c.395A>C	1	1	132	p.Gln132Pro	5
c.402_405del	1	1	134	p.Glu134Aspfs*24	5
c.408delT	1	1	136	p.Phe136Leufs*23	5
c.412C>A	1	1	138	p.Pro138Thr	4
c.422_440del	1	1	141	p.Asn141Ilefs*12	5
c.447delA	1	1	150	p.Asn150Ilefs*9	4
c.449_462del	1	1	150	p.Asn150Serfs*19	4
c.451+452dupl.AT(451_552del)	1	1	151	Frameshift	4
c.453C>G	1	1	151	p.Ile151Met	4
c.456_457del	1	1	153	p.Leu153Alafs*20	4
c.458T>A	1	1	153	p.Leu153Gln	4
c.460C>A	1	1	154	p.Pro154Thr	4
c.462A>C	1	1	154	p.Pro154=	4

c.463G>C	1	1	155	p.Val155Leu	5
c.463G>T	1	1	155	p.Val155Leu	5
c.464T>G	1	1	155	p.Val155Gly	5
c.474insA	1	1	158	p.Glu160Argfs*14	5
c.475A>G	1	1	159	p.Lys159Glu	5
c.479A>T	1	1	160	p.Glu160Val	4
c.485G>T	1	1	162	p.Cys162Phe	5
c.487C>T	1	1	163	p.Leu163Phe	5
c.487delC	1	1	163	p.Leu163Serfs*7	4
c.491A>G	1	1	164	p.Gln164Arg	5
c.492G>C	1	1	164	p.Gln164His	5
c.493G>T	1	1	165	p.Val165Trp	4
c.499_504delinsT	1	1	167	p.Arg167Serfs*5	4
c.499C>G	1	1	167	p.Arg167Gly	5
c.500G>T	1	1	167	p.Arg167Leu	5
c.506T>C	1	1	169	p.Leu169Pro	5
c.509T>C	1	1	170	p.Val170Ala	4
c.523T>A	1	1	175	p.Tyr175Asn	4
c.524dupA	1	1	175	p.Tyr175Ter	5
c.527delG	1	1	177	p.Arg177Aspfs*25	5
c.529A>T	1	1	177	p.Arg177Ter	5
c.533_534del	1	1	178	p.Leu178Argfs*77	5
c.548C>G	1	1	183	p.Ser183Trp	4
c.555C>A	1	1	185	p.Tyr185Ter	5
c.572_574delACCHis191del	1	1	191	p.His191del	4
c.573delC	1	1	192	p.Pro192Glnfs*10	5
c.588_588dupA	1	1	197	p.Asp197Argfs*59	4
c.589G>A	1	1	197	p.Asp197Asn	5
c.640T>A	1	1	214	p.Ter214ArgexTer14	4
c.340+1G>A	1	1	NA	p.?	5
c.341-2A>C	1	1	NA	p.?	4

ACMG class: variant class (4: likely pathogenic, 5: pathogenic) according to American College of Medical Genetics and Genomics (ACMG).

Mutations highlighted in bold were observed in ≥ 30 participants.

Supplementary Table 3: Results of pairwise comparisons of age-related penetrance curves and of Cox regression per manifestation.

	Mutation 1		Mutation 2		Logrank Test				Cox regression with mutation 1 as reference		Cox regression with mutation 2 as reference	
Manifestation	Mutation	Frequency carriers	Mutation	Frequency carriers	p	Significance category	p.adjust	Significance category	HR	95% CI	HR	95% CI
Retinal hemangioblastoma	p.Arg161Gln	43	p.Arg161Ter	41	4,19E-02	p<0.05*	8,03E-02	p>0.05	1,98	(1;3.93)	0,51	(0.25;1)
Retinal hemangioblastoma	p.Arg161Gln	43	p.Arg167Gln	68	9,17E-01	p>0.05	9,44E-01	p>0.05	0,92	(0.47;1.79)	1,09	(0.56;2.11)
Retinal hemangioblastoma	p.Arg161Gln	43	p.Arg167Trp	84	8,44E-01	p>0.05	9,34E-01	p>0.05	0,91	(0.47;1.75)	1,10	(0.57;2.11)
Retinal hemangioblastoma	p.Arg161Gln	43	p.Asn78Ser	30	8,72E-01	p>0.05	9,34E-01	p>0.05	0,96	(0.45;2.04)	1,05	(0.49;2.23)
Retinal hemangioblastoma	p.Arg161Gln	43	p.Tyr98His	214	2,24E-01	p>0.05	3,36E-01	p>0.05	0,73	(0.41;1.32)	1,36	(0.76;2.45)
Retinal hemangioblastoma	p.Arg161Ter	41	p.Arg167Gln	68	1,39E-02	p<0.05*	3,12E-02	p<0.05*	0,46	(0.26;0.82)	2,15	(1.22;3.79)
Retinal hemangioblastoma	p.Arg161Ter	41	p.Arg167Trp	84	8,09E-03	p<0.01**	2,02E-02	p<0.05*	0,46	(0.26;0.8)	2,17	(1.25;3.79)
Retinal hemangioblastoma	p.Arg161Ter	41	p.Asn78Ser	30	1,10E-01	p>0.05	1,97E-01	p>0.05	0,48	(0.25;0.95)	2,07	(1.06;4.06)
Retinal hemangioblastoma	p.Arg161Ter	41	p.Tyr98His	214	1,76E-05	p<0.0001****	6,08E-05	p<0.05*	0,37	(0.23;0.59)	2,70	(1.68;4.32)
Retinal hemangioblastoma	p.Arg167Gln	68	p.Arg167Trp	84	9,34E-01	p>0.05	9,44E-01	p>0.05	0,99	(0.59;1.68)	1,01	(0.6;1.71)
Retinal hemangioblastoma	p.Arg167Gln	68	p.Asn78Ser	30	8,65E-01	p>0.05	9,34E-01	p>0.05	1,04	(0.55;1.99)	0,96	(0.5;1.83)
Retinal hemangioblastoma	p.Arg167Gln	68	p.Tyr98His	214	2,77E-01	p>0.05	4,00E-01	p>0.05	0,80	(0.52;1.22)	1,25	(0.82;1.92)
Retinal hemangioblastoma	p.Arg167Trp	84	p.Asn78Ser	30	9,63E-01	p>0.05	9,63E-01	p>0.05	1,05	(0.55;1.99)	0,95	(0.5;1.8)
Retinal hemangioblastoma	p.Arg167Trp	84	p.Tyr98His	214	2,38E-01	p>0.05	3,51E-01	p>0.05	0,81	(0.53;1.22)	1,24	(0.82;1.88)
Retinal hemangioblastoma	p.Asn78Ser	30	p.Tyr98His	214	3,38E-01	p>0.05	4,76E-01	p>0.05	0,77	(0.44;1.34)	1,30	(0.75;2.28)
Intracranial hemangioblastoma	p.Arg161Gln	42	p.Arg161Ter	42	5,60E-04	p<0.001***	1,57E-03	p<0.05*	3,89	(1.68;8.99)	0,26	(0.11;0.6)
Intracranial hemangioblastoma	p.Arg161Gln	42	p.Arg167Gln	67	1,05E-02	p<0.05*	2,46E-02	p<0.05*	2,54	(1.13;5.74)	0,39	(0.17;0.89)
Intracranial hemangioblastoma	p.Arg161Gln	42	p.Arg167Trp	83	1,57E-01	p>0.05	2,56E-01	p>0.05	1,83	(0.8;4.19)	0,55	(0.24;1.25)
Intracranial hemangioblastoma	p.Arg161Gln	42	p.Asn78Ser	30	1,22E-03	p<0.01**	3,34E-03	p<0.05*	3,67	(1.58;8.53)	0,27	(0.12;0.63)
Intracranial hemangioblastoma	p.Arg161Gln	42	p.Tyr98His	207	3,43E-02	p<0.05*	6,94E-02	p>0.05	0,52	(0.23;1.17)	1,92	(0.85;4.3)
Intracranial hemangioblastoma	p.Arg161Ter	42	p.Arg167Gln	67	1,45E-01	p>0.05	2,50E-01	p>0.05	0,65	(0.39;1.1)	1,53	(0.91;2.56)
Intracranial hemangioblastoma	p.Arg161Ter	42	p.Arg167Trp	83	3,60E-03	p<0.01**	9,27E-03	p<0.05*	0,47	(0.27;0.81)	2,12	(1.24;3.64)
Intracranial hemangioblastoma	p.Arg161Ter	42	p.Asn78Ser	30	9,34E-01	p>0.05	9,44E-01	p>0.05	0,95	(0.54;1.66)	1,06	(0.6;1.86)
Intracranial hemangioblastoma	p.Arg161Ter	42	p.Tyr98His	207	0,00E+00	p<0.0001****	0,00E+00	p<0.05*	0,13	(0.08;0.22)	7,44	(4.49;12.35)
Intracranial hemangioblastoma	p.Arg167Gln	67	p.Arg167Trp	83	1,43E-01	p>0.05	2,50E-01	p>0.05	0,72	(0.44;1.19)	1,39	(0.84;2.29)
Intracranial hemangioblastoma	p.Arg167Gln	67	p.Asn78Ser	30	2,19E-01	p>0.05	3,36E-01	p>0.05	1,44	(0.85;2.44)	0,69	(0.41;1.17)
Intracranial hemangioblastoma	p.Arg167Gln	67	p.Tyr98His	207	1,89E-14	p<0.0001****	1,54E-13	p<0.05*	0,21	(0.13;0.33)	4,87	(3.07;7.73)
Intracranial hemangioblastoma	p.Arg167Trp	83	p.Asn78Ser	30	1,07E-02	p<0.05*	2,46E-02	p<0.05*	2,00	(1.16;3.46)	0,50	(0.29;0.86)
Intracranial hemangioblastoma	p.Arg167Trp	83	p.Tyr98His	207	7,79E-09	p<0.0001****	4,38E-08	p<0.05*	0,28	(0.17;0.47)	3,51	(2.15;5.74)
Intracranial hemangioblastoma	p.Asn78Ser	30	p.Tyr98His	207	0,00E+00	p<0.0001****	0,00E+00	p<0.05*	0,14	(0.09;0.24)	7,04	(4.21;11.75)
Spinal hemangioblastoma	p.Arg161Gln	42	p.Arg161Ter	42	3,47E-02	p<0.05*	6,94E-02	p>0.05	2,14	(0.99;4.66)	0,47	(0.21;1.01)
Spinal hemangioblastoma	p.Arg161Gln	42	p.Arg167Gln	67	9,27E-01	p>0.05	9,44E-01	p>0.05	0,93	(0.43;2.02)	1,08	(0.49;2.34)
Spinal hemangioblastoma	p.Arg161Gln	42	p.Arg167Trp	83	7,28E-01	p>0.05	8,50E-01	p>0.05	1,14	(0.54;2.4)	0,88	(0.42;1.86)
Spinal hemangioblastoma	p.Arg161Gln	42	p.Asn78Ser	30	3,63E-01	p>0.05	4,89E-01	p>0.05	1,40	(0.62;3.15)	0,71	(0.32;1.6)
Spinal hemangioblastoma	p.Arg161Gln	42	p.Tyr98His	202	4,73E-01	p>0.05	6,08E-01	p>0.05	0,87	(0.44;1.74)	1,15	(0.58;2.29)
Spinal hemangioblastoma	p.Arg161Ter	42	p.Arg167Gln	67	9,78E-03	p<0.01**	2,38E-02	p<0.05*	0,43	(0.24;0.78)	2,31	(1.28;4.17)
Spinal hemangioblastoma	p.Arg161Ter	42	p.Arg167Trp	83	1,72E-02	p<0.05*	3,68E-02	p<0.05*	0,53	(0.3;0.92)	1,89	(1.08;3.29)
Spinal hemangioblastoma	p.Arg161Ter	42	p.Asn78Ser	30	3,64E-01	p>0.05	4,89E-01	p>0.05	0,66	(0.35;1.24)	1,53	(0.81;2.88)
Spinal hemangioblastoma	p.Arg161Ter	42	p.Tyr98His	202	6,52E-05	p<0.0001****	2,02E-04	p<0.05*	0,41	(0.25;0.65)	2,46	(1.54;3.93)
Spinal hemangioblastoma	p.Arg167Gln	67	p.Arg167Trp	83	5,99E-01	p>0.05	7,39E-01	p>0.05	1,22	(0.7;2.13)	0,82	(0.47;1.43)
Spinal hemangioblastoma	p.Arg167Gln	67	p.Asn78Ser	30	1,64E-01	p>0.05	2,64E-01	p>0.05	1,51	(0.8;2.85)	0,66	(0.35;1.25)
Spinal hemangioblastoma	p.Arg167Gln	67	p.Tyr98His	202	6,59E-01	p>0.05	8,01E-01	p>0.05	0,94	(0.59;1.5)	1,07	(0.67;1.7)
Spinal hemangioblastoma	p.Arg167Trp	83	p.Asn78Ser	30	5,07E-01	p>0.05	6,43E-01	p>0.05	1,24	(0.68;2.25)	0,81	(0.44;1.47)
Spinal hemangioblastoma	p.Arg167Trp	83	p.Tyr98His	202	1,53E-01	p>0.05	2,55E-01	p>0.05	0,77	(0.5;1.17)	1,30	(0.85;1.98)
Spinal hemangioblastoma	p.Asn78Ser	30	p.Tyr98His	202	4,09E-02	p<0.05*	8,00E-02	p>0.05	0,62	(0.37;1.05)	1,61	(0.96;2.71)
Renal cell carcinoma	p.Arg161Gln	42	p.Arg161Ter	42	2,08E-05	p<0.0001****	6,94E-05	p<0.05*	6,43	(2.43;17)	0,16	(0.06;0.41)
Renal cell carcinoma	p.Arg161Gln	42	p.Arg167Gln	67	1,47E-01	p>0.05	2,50E-01	p>0.05	2,12	(0.8;5.61)	0,47	(0.18;1.24)
Renal cell carcinoma	p.Arg161Gln	42	p.Arg167Trp	83	8,25E-01	p>0.05	9,28E-01	p>0.05	1,15	(0.42;3.21)	0,87	(0.31;2.41)
Renal cell carcinoma	p.Arg161Gln	42	p.Asn78Ser	30	2,84E-03	p<0.01**	7,52E-03	p<0.05*	3,95	(1.46;10.64)	0,25	(0.09;0.68)
Renal cell carcinoma	p.Arg161Gln	42	p.Tyr98His	217	4,36E-06	p<0.0001****	1,70E-05	p<0.05*	0,08	(0.02;0.28)	12,57	(3.57;44.19)
Renal cell carcinoma	p.Arg161Ter	42	p.Arg167Gln	67	1,92E-04	p<0.001***	5,75E-04	p<0.05*	0,33	(0.18;0.6)	3,03	(1.68;5.47)
Renal cell carcinoma	p.Arg161Ter	42	p.Arg167Trp	83	1,85E-08	p<0.0001****	9,79E-08	p<0.05*	0,18	(0.09;0.35)	5,57	(2.85;10.9)
Renal cell carcinoma	p.Arg161Ter	42	p.Asn78Ser	30	2,80E-01	p>0.05	4,00E-01	p>0.05	0,61	(0.33;1.15)	1,63	(0.87;3.05)
Renal cell carcinoma	p.Arg161Ter	42	p.Tyr98His	217	0,00E+00	p<0.0001****	0,00E+00	p<0.05*	0,01	(0;0.03)	80,79	(29.58;220.65)
Renal cell carcinoma	p.Arg167Gln	67	p.Arg167Trp	83	6,29E-02	p>0.05	1,18E-01	p>0.05	0,54	(0.28;1.06)	1,84	(0.94;3.59)
Renal cell carcinoma	p.Arg167Gln	67	p.Asn78Ser	30	6,52E-02	p>0.05	1,20E-01	p>0.05	1,86	(1;3.46)	0,54	(0.29;1)
Renal cell carcinoma	p.Arg167Gln	67	p.Tyr98His	217	0,00E+00	p<0.0001****	0,00E+00	p<0.05*	0,04	(0.01;0.1)	26,68	(10.02;71.08)
Renal cell carcinoma	p.Arg167Trp	83	p.Asn78Ser	30	3,39E-04	p<0.001***	9,85E-04	p<0.05*	3,42	(1.7;6.88)	0,29	(0.15;0.59)
Renal cell carcinoma	p.Arg167Trp	83	p.Tyr98His	217	5,67E-11	p<0.0001****	3,64E-10	p<0.05*	0,07	(0.02;0.2)	14,50	(5.11;41.16)
Renal cell carcinoma	p.Asn78Ser	30	p.Tyr98His	217	0,00E+00	p<0.0001****	0,00E+00	p<0.05*	0,02	(0.01;0.06)	49,58	(18;136.56)
Pancreatic neuroendocrine tumor	p.Arg161Gln	42	p.Arg161Ter	42	8,61E-01	p>0.05	9,34E-01	p>0.05	0,91	(0.36;2.31)	1,09	(0.43;2.76)
Pancreatic neuroendocrine tumor	p.Arg161Gln	42	p.Arg167Gln	67	8,11E-01	p>0.05	9,28E-01	p>0.05	1,15	(0.54;2.47)	0,87	(0.41;1.86)
Pancreatic neuroendocrine tumor	p.Arg161Gln	42	p.Arg167Trp	83	8,86E-01	p>0.05	9,38E-01	p>0.05	1,05	(0.49;2.25)	0,95	(0.45;2.04)
Pancreatic neuroendocrine tumor	p.Arg161Gln	42	p.Asn78Ser	30	3,71E-01	p>0.05	4,90E-01	p>0.05	0,67	(0.26;1.74)	1,49	(0.57;3.89)
Pancreatic neuroendocrine tumor	p.Arg161Gln	42	p.Tyr98His	217	3,33E-16	p<0.0001****	3,33E-15	p<0.05*	0,04	(0.01;0.12)	25,66	(8.44;78.02)
Pancreatic neuroendocrine tumor	p.Arg161Ter	42	p.Arg167Gln	67	5,27E-01	p>0.05	6,58E-01	p>0.05	1,26	(0.59;2.7)	0,79	(0.37;1.7)
Pancreatic neuroendocrine tumor	p.Arg161Ter	42	p.Arg167Trp	83	7,11E-01	p>0.05	8,42E-01	p>0.05	1,15	(0.54;2.46)	0,87	(0.41;1.87)
Pancreatic neuroendocrine tumor	p.Arg161Ter	42	p.Asn78Ser	30	4,30E-01	p>0.05	5,62E-01	p>0.05	0,73	(0.28;1.91)	1,37	(0.52;3.56)
Pancreatic neuroendocrine tumor	p.Arg161Ter	42	p.Tyr98His	217	2,77E-12	p<0.0001****	1,91E-11	p<0.05*	0,04	(0.01;0.13)	23,47	(7.7;71.54)
Pancreatic neuroendocrine tumor	p.Arg167Gln	67	p.Arg167Trp	83	6,96E-01	p>0.05	8,35E-01	p>0.05	0,91	(0.53;1.56)	1,10	(0.64;1.89)
Pancreatic neuroendocrine tumor	p.Arg167Gln	67	p.Asn78Ser	30	2,04E-01	p>0.05	3,22E-01	p>0.05	0,58	(0.26;1.28)	1,72	(0.78;3.8)
Pancreatic neuroendocrine tumor	p.Arg167Gln	67	p.Tyr98His	217	0,00E+00	p<0.0001****	0,00E+00	p<0.05*	0,03	(0.01;0.09)	29,58	(11.31;77.36)
Pancreatic neuroendocrine tumor	p.Arg167Trp	83	p.Asn78Ser	30	2,21E-01	p>0.05	3,36E-01	p>0.05	0,64	(0.29;1.41)	1,57	(0.71;3.47)
Pancreatic neuroendocrine tumor	p.Arg167Trp	83	p.Tyr98His	217	0,00E+00	p<0.0001****	0,00E+00	p<0.05*	0,04	(0.01;0.1)	26,94	(10.21;71.08)
Pancreatic neuroendocrine tumor	p.Asn78Ser	30	p.Tyr98His	217	2,22E-10	p<0.0001****	1,33E-09	p<0.05*	0,06	(0.02;0.18)	17,18	(5.56;53.03)
Pheochromocytoma/ paraganglioma	p.Arg161Gln	42	p.Arg161Ter	42	1,29E-14	p<0.0001****	1,16E-13	p<0.05*	0,05	(0.02;0.13)	18,93	(7.46;48.02)
Pheochromocytoma/ paraganglioma	p.Arg161Gln	42	p.Arg167Gln	68	3,01E-07	p<0.0001****	1,43E-06	p<0.05*	0,33	(0.21;0.5)	3,06	(1.99;4.68)
Pheochromocytoma/ paraganglioma	p.Arg161Gln	42	p.Arg167Trp	83	7,28E-07	p<0.0001****	3,28E-06	p<0.05*	0,36	(0.24;0.54)	2,76	(1.84;4.15)
Pheochromocytoma/ paraganglioma	p.Arg161Gln	42	p.Asn78Ser	29	1,17E-12	p<0.0001****	8,75E-12	p<0.05*	0,05	(0.02;0.13)	21,33	(7.61;59.79)
Pheochromocytoma/ paraganglioma	p.Arg161Gln	42	p.Tyr98His	218	0,00E+00	p<0.0001****	0,00E+00	p<0.05*	0,15	(0.1;0.21)	6,81	(4.67;9.95)
Pheochromocytoma/ paraganglioma	p.Arg161Ter	42	p.Arg167Gln	68	1,64E-05	p<0.0001****	5,92E-05	p<0.05*	6,19	(2.46;15.59)	0,16	(0.06;0.41)
Pheochromocytoma/ paraganglioma	p.Arg161Ter	42	p.Arg167Trp	83	2,36E-06	p<0.0001****	1,01E-05	p<0.05*	6,85	(2.74;17.09)	0,15	(0.06;0.36)
Phaeochromocytoma/ paraganglioma	p.Arg161Ter	42	p.Asn78Ser	29	8,19E-01	p>0.05	9,28E-01	p>0.05	0,89	(0.24;3.31)	1,13	(0.3;4.2)
Pheochromocytoma/ paraganglioma	p.Arg161Ter	42	p.Tyr98His	218	1,89E-02	p<0.05*	3,95E-02	p<0.05*	2,78	(1.13;6.84)	0,36	(0.15;0.89)
Phaeochromocytoma/ paraganglioma	p.Arg167Gln	68	p.Arg167Trp	83	3,62E-01	p>0.05	4,89E-01	p>0.05	1,11	(0.75;1.63)	0,90	(0.61;1.33)
Pheochromocytoma/ paraganglioma	p.Arg167Gln	68	p.Asn78Ser	29	4,20E-05	p<0.0001****	1,35E-04	p<0.05*	0,14	(0.05;0.4)	6,98	(2.51;19.41)
Pheochromocytoma/ paraganglioma	p.Arg167Gln	68	p.Tyr98His	218	4,95E-06	p<0.0001****	1,85E-05	p<0.05*	0,45	(0.32;0.64)	2,23	(1.57;3.17)
Pheochromocytoma/ paraganglioma	p.Arg167Trp	83	p.Asn78Ser	29	3,99E-06	p<0.0001****	1,63E-05	p<0.05*	0,13	(0.05;0.36)	7,72	(2.8;21.29)
Pheochromocytoma/ paraganglioma	p.Arg167Trp	83	p.Tyr98His	218	6,12E-08	p<0.0001****	3,06E-07	p<0.05*	0,41	(0.29;0.57)	2,47	(1.77;3.43)
Pheochromocytoma/ paraganglioma	p.Asn78Ser	29	p.Tyr98His	218	1,52E-02	p<0.05*	3,34E-02	p<0.05*	3,13	(1.15;8.51)	0,32	(0.12;0.87)

Logrank test was conducted to pairwise compare penetrance curves of different *VHL* mutations per tumor manifestation.

For convenience, reported p-values were categorized into (Significance category): p <0.0001****, <0.001***, <0.01**, <0.05*, >0.05

P: uncorrected p-value of logrank test, p.adjust: corrected p-value of logrank test using Benjamini-Hochberg approach to correct for multiple testing.

Cox regression was performed to estimate the instantenous risk of one mutation in comparison to a reference mutation.

For convenience, this analysis was conducted twice by exchanging the reference mutation for a given pair of mutations.

HR: hazard ratio, 95% CI: 95% confidence interval.

Supplementary Table 4: List of *VHL* germline variants with PPGL only (type 2C) phenotype

VHL mutation	Frequency carriers	Frequency contributing centers	Codon	VHL protein variant	Carriers with PPGL only (type 2C) phenotype (Number,%)	ACMG class
**c.292T>C**	**226**	**5**	**98**	**p.Tyr98His**	**28 (12.4)**	**5**
**c.499C>T**	**84**	**18**	**167**	**p.Arg167Trp**	**14 (16.7)**	**5**
**c.500G>A**	**68**	**21**	**167**	**p.Arg167Gln**	**5 (7.4)**	**5**
**c.482G>A**	**43**	**10**	**161**	**p.Arg161Gln**	**19 (44.2)**	**5**
c.227-229delTCT	22	6	76	p.Phe76del	1 (4.5)	5
c.277G>A	13	4	93	p.Gly93Ser	6 (46.2)	5
c.467A>G	13	4	156	p.Tyr156Cys	6 (46.2)	5
c.562C>G	12	3	188	p.Leu188Val	4 (33.3)	5
c.575C>T	12	1	192	p.Pro192Leu	9 (75.0)	4
c.599G>C	11	1	200	p.Arg200Pro	6 (54.5)	4
c.191G>C	10	5	64	p.Arg64Pro	5 (50.0)	5
c.256C>G	10	3	86	p.Pro86Ala	1 (10.0)	5
c.481C>G	10	3	161	p.Arg161Gly	2 (20.0)	5
c.256C>T	9	4	86	p.Pro86Ser	3 (33.3)	5
c.320G>A	9	1	107	p.Arg107His	1 (11.1)	5
c.593T>A	8	1	198	p.Leu198Gln	1 (12.5)	5
c.340G>A	8	1	114	p.Gly114Ser	2 (25.0)	5
c.496G>T	8	5	166	p.Val166Phe	1 (12.5)	5
c.640T>G	5	2	214	p.Ter214GlyextTer14	1 (20.0)	4
c.241C>T	5	3	81	p.Pro81Ser	1 (20.0)	4
c.244C>G	5	1	82	p.Arg82Gly	3 (60.0)	4
c.593T>C	5	3	198	p.Leu198Pro	4 (80.0)	4
c.250G>T	4	2	84	p.Val84Leu	4 (100.0)	5
c.319C>G	4	3	107	p.Arg107Gly	2 (56.0)	5
c.335A>G	3	2	112	p.Tyr112Cys	2 (66.7)	5
c.353T>G	3	1	118	p.Leu118Arg	1 (33.3)	5
c.488T>G	3	2	163	p.Leu163Arg	2 (66.7)	5
c.250G>C	2	2	84	p.Val84Leu	1 (50.0)	5
c.388G>A	2	1	130	p.Val130Ile	1 (50.0)	4
c.389T>G	2	1	130	p.Val130Gly	1 (50.0)	5
c.283C>G	1	1	95	p.Pro95Ala	1 (100.0)	4
c.308C>A	1	1	103	p.Pro103His	1 (100.0)	4
c.334T>C	1	1	112	p.Tyr112His	1 (100.0)	5
c.360A>C	1	1	120	p.Arg120Ser	1 (100.0)	4
c.458T>A	1	1	153	p.Leu153Gln	1 (100.0)	4
c.487C>T	1	1	163	p.Leu163Phe	1 (100.0)	5
c.491A>G	1	1	164	p.Gln164Arg	1 (100.0)	5
c.492G>C	1	1	164	p.Gln164His	1 (100.0)	5
c.509T>C	1	1	170	p.Val170Ala	1 (100.0)	4
c.548C>G	1	1	183	p.Ser183Trp	1 (100.0)	4
c.572_574delACCHis191del	1	1	191	p.His191del	1 (100.0)	4
c.588_588dupA	1	1	197	p.Asp197Argfs*59	1 (100.0)	4
c.640T>A	1	1	214	p.Ter214ArgexTer14	1 (100.0)	4

ACMG class: variant class (4: likely pathogenic, 5: pathogenic) according to American College of Medical Genetics and Genomics (ACMG).

Mutations highlighted in bold were observed in ≥ 30 participants.
